# Parent-child relationship in children of alcoholic and non-alcoholic parents

**DOI:** 10.4103/0972-6748.57855

**Published:** 2009

**Authors:** Babita Mahato, Arif Ali, Masroor Jahan, A. N. Verma, Amool R. Singh

**Affiliations:** 1 Department of Psychiatric Social Work, RINPAS, Kanke, Ranchi, India; 2 LGBRIMH Tezpur Assam, India; 3 Department of Clinical Psychology, RINPAS, Kanke, Ranchi, India

**Keywords:** Children of alcoholics and non-alcoholics, Parent-child relationship, Dimensions of parenting

## Abstract

**Aim:**

Overall aim of the study was to see parent-child relationship in children of alcoholic and non-alcoholic parents.

**Materials and Methods:**

The sample consisted of 30 alcoholic and 30 non-alcoholic parents and their children taken from Kanke Block of Ranchi district. The sample was selected on the basis of inclusion and exclusion criteria. Socio-demographic data sheet and Parent Child Relationship Scale (Rao, 1978) were administered to the children.

**Results:**

In a child’s perception of father in various domains of parent-child relationship, significant difference at *P* < 0.01 was found in the domain of symbolic punishment, rejecting, objective punishment, demanding, indifferent, symbolic reward in loving and neglecting, and in child’s perception of the mother. Significant difference at *P* < 0.01 was found in the domain of symbolic punishment, rejecting, object punishment, indifferent and in neglecting.

**Conclusion:**

The result showed that the children of alcoholic parents tended to have more symbolic punishment, rejecting, objective punishment, demanding, indifferent, symbolic reward loving and in neglecting than children of non alcoholic parents.

An alcoholic family’s home environment and the manner in which family members interact may contribute to the risk of the problems observed among children of alcoholics. Although alcoholic families are a heterogeneous group, some common characteristics have been identified. Families of alcoholics have lower levels of family cohesion, expressiveness, independence, and intellectual orientation and higher levels of conflict compared with non-alcoholic families (Filstead *et al*., 1981; Moos & Billings, 1982; Moos & Moos, 1984; Clair & Genest, 1986). Some characteristics, however, are not specific to alcoholic families. Impaired problem-solving ability and hostile communication are observed both in alcoholic families and in families with problems other than alcohol (Billings *et al*., 1979). Moreover, the characteristics of families with recovering alcoholic members and of families with no alcoholic members do not differ significantly, suggesting that a parent’s continued drinking may be responsible for the disruption of family life in an alcoholic home (Moos & Billings, 1982). Studies comparing children of alcoholics with those of non-alcoholics have also found that parental alcoholism is linked to a number of psychological disorders in children. Divorce, parental anxiety or affective disorders, or undesirable changes in the family or in life situations can add to the negative effect of parental alcoholism on children’s emotional functioning (Schuckit & Chiles, 1978; Moos & Billings, 1982). A number of influential clinicians (Black, 1982) have described children of alcoholics as victims of an alcoholic family environment characterized by disruption, deviant parental role models, inadequate parenting, and disturbed parent-child relationships. These family-related variables are thought to undermine normal psychological development and to cause distress and impaired interpersonal functioning, both acutely and chronically. In a study conducted on the effects of alcohol on parents’ interactions with children, it was found that parents are unable to respond appropriately to a child’s improper behavior. Although the child is acting improperly, the group of intoxicated parents not only fails to discipline the child, but engage in parental indulgences that are inappropriate for the occasion (Lang *et al*., 1999). Eiden *et al*. (2004) examined the transactional nature of parent-child interactions over time among alcoholic and non-alcoholic families. They found that long-term alcohol intake was predictive of negative parental behavior. Kearns-Bodkin and Leonard (2008) suggested that children raised in alcoholic families may carry the problematic effects of their early family environment into their adult relationships. Hence, parent-child relationship is very important while working with children of alcoholic parents. Keeping this point in view, the present study aimed to assess parent-child relationship in children of alcoholic and non-alcoholic parents.

## MATERIALS AND METHODS

### Sample

The sample consisted of 30 alcoholic and 30 non-alcoholic parents from the Kanke Block of Ranchi district. The age range of parents was 25 to 50 years. Those parents were included who had history of more than five years of alcohol abuse and who were interacting with their children for at least five years. They had minimum primary level of educational qualification and gave consent to participate in the study. Parents having multiple substance abuse and co-morbid psychiatric illness were excluded.

Child participants were between the age range of 12 and 17 years. Only eldest children who did not have history of developmental delay were taken. Mean age of children of alcoholic parents was 13.50 ± 1.13 years, and of children of non-alcoholic parents was 3.96 ± 1.51 years. Socio-demographic characteristics of children of alcoholic and non-alcoholic parents are given in Table 1.

**Table 1:** Socio-demographic characteristics of children

Variables	Group 1 - Children of alcoholic parents N = 30		Group 2 - Children of non-alcoholic parents N = 30		Value of Chi square (df)
	Frequency	%	Frequency	%	
Religion					5.200 (3)
Hindu	25	83.3	25	83.3	
Muslim	3	10.0	2	6.7	
Christian	0	0.0	3	10.0	
Others	2	6.7	0	0.0	
Caste					3.486 (3)
General	7	23.3	9	30.0	
SC	3	10.0	0	0.0	
ST	5	16.7	4	13.3	
OBC	15	50.0	17	56.7	
Education					16.952** (3)
Primary	2	6.7	5	16.7	
Secondary	28	93.3	14	46.7	
Matriculation and above	0	0.0	11	33.3	
Intermediate	0	0.0	1	3.3	
Family income					3.309 (2)
Up to 5000	22	73.3	21	70.0	
Up to 10000	8	26.7	6	20.0	
Above 10000	0	0.0	3	10.0	

** *P* < .01

### Tools

#### Socio-demographic data sheet

Socio-demographic data was prepared to obtain background information about the subject on dimensions like age, sex, marital status, education, income, residential area, type of family etc.

#### Parent-child relationship scale (PCRS; Rao, 1989)

The present scale is adapted from the revised Roe-Seigalman parent-child relationship questionnaire that measures the characteristic behavior of parents as experienced by their children. It consists of 100 items categorized into ten dimensions, i.e., protecting, symbolic, punishment, rejecting, object punishment, demanding, indifferent, symbolic reward, loving, object reward and neglecting. At first all the selected participants were contacted individually and consent was taken to participate in the study. They were informed regarding purpose of the research. First, socio-demographic details were taken from parents and Parent-Child Relationship Scale (PCRS) was administered to the children. The Statistical Package for Social Sciences (SPSS), Version 13.0 was used for the analysis of the data. Percentage, chi-square test, *t*-test, and correlation were used to analyze the data.

## RESULT

The sample consisted of a total of 60 participants; 30 children of alcoholic and 30 children of non-alcoholic parents. Table 2 shows mean and standard deviation (SD) of scores obtained by children of alcoholic and non-alcoholic parents in different domains of PCRS towards father. Significant difference was found in the domains of symbolic punishment, rejecting, objective punishment, demanding, indifferent, symbolic reward, loving and neglecting.

**Table 2:** Child’s perception of father in various domains of parent-child relationship

Variable	Children of alcoholic N = 30		Children of non-alcoholic N = 30		*t*
	Mean	SD	Mean	SD	
Protecting	46.10	10.06	46.16	10.04	0.026
Symbolic punishment	40.30	10.73	22.26	5.57	8.167**
Rejecting	37.80	11.91	26.60	4.51	4.816**
Object punishment	35.03	10.67	26.33	7.35	3.677**
Demanding	38.53	11.00	28.40	6.04	4.419**
Indifferent	35.86	9.95	30.13	5.69	2.739**
Symbolic reward	44.66	10.37	31.36	5.97	6.084**
Loving	45.56	10.46	30.66	5.22	6.975**
Object reward	37.83	10.12	31.96	7.43	2.558
Neglecting	35.60	9.78	29.03	4.29	3.365**

** *P* < 0.01

Table 3 shows the mean and standard deviation of scores obtained by children of alcoholic and non-alcoholic parents in various domains of PCRS towards mother. Significant difference was found in the domains of symbolic punishment, rejecting, object punishment, indifferent, neglecting and demanding. Correlation between various domains of parent-child relationship and duration of alcohol intake was done in the children of alcoholic parents.

**Table 3:** Child’s perception of mother in various domains of parent-child relationship

Variable	Children of alcoholic N = 30		Children of non-alcoholic N = 30		*t*
	Mean	SD	Mean	SD	
Protecting	35.56	7.52	33.40	3.94	1.396
Symbolic punishment	24.83	4.76	28.13	4.49	2.760**
Rejecting	21.20	4.89	29.33	4.68	6.575**
Object punishment	19.26	4.20	26.63	5.55	5.789**
Demanding	25.53	5.07	28.66	5.69	2.251*
Indifferent	25.30	5.96	29.43	5.64	2.757**
Symbolic reward	32.53	5.85	30.63	3.75	1.496
Loving	32.93	7.75	32.06	3.75	0.551
Object reward	25.63	7.39	27.70	5.19	1.253
Neglecting	22.80	7.31	28.93	4.34	−3.947**

* *P* < 0.05

** *P* < 0.01

There was significant correlation between neglecting and object reward with duration of alcohol intake [Table 4].

**Table 4:** Correlation between various domains of parent-child relationship and duration of alcohol intake

Variable	Protecting	Symbolic punishment	Rejecting	Object punishment	Demanding	Indifferent	Symbolic reward	Loving	Object reward	Neglecting
Duration of alcohol intake	0.260	0.249	0.233	0.282	0.246	0.269	0.278	0.311	0.371*	0.381*

* *P* < 0.05

## DISCUSSION

The present study was conducted with the aim of comparing the parent-child relationship in children of alcoholic and non-alcoholic parents. In parent-child relationship a significant difference was found in the domains of symbolic punishment, rejecting, objective punishment, demanding, indifferent, symbolic reward. loving, and neglecting for father. In child’s relationship with mother, significant difference was found in the domain of symbolic punishment, rejecting, object punishment, indifferent and neglecting.

Previous studies also reported that families of alcoholics have lower levels of family cohesion, expressiveness, independence, and intellectual orientation and higher levels of conflict compared with non-alcoholic families. Some characteristics, however, are not specific to alcoholic families: Impaired problem-solving ability and hostile communication are observed both in alcoholic families and in families with problems other than alcohol (Billings *et al*., 1979). Alcoholic parents have a negative effect on their children because the effect of alcohol undermines their capacity to use their parenting skills in a number of ways. First, excessive drinking by the parents can lead to inconsistent parenting behavior. When the child misbehaves in a certain way, the parents may overreact by screaming at the child on one occasion; on another occasion, parents may act indulgently towards the child. Consequently, the child receives mixed signals about appropriate behavior. In addition, the inconsistency in parenting behaviors creates an unpredictable and unstable environment that can undermine the child’s mental and emotional growth (Windle, 1996). Haugland (2003) examined possible risk factors associated with child adjustment in a sample of children with alcohol-abusing fathers. Factors included were socioeconomic status, severity of the fathers’ alcohol abuse, parental psychological problems, and family functioning. The finding further suggested that child adjustment in families with paternal alcohol abuse is the result of an accumulation of risk factors rather than the effects of the paternal alcohol abuse alone. Both, general environmental risk factors (psychological problems in the fathers, family climate, family health and conflicts) and environmental factors related to the parental alcohol abuse (severity of the alcohol abuse, the child’s level of exposure to the alcohol abuse, changes in routines and rituals due to drinking) were related to child adjustment. The result indicated the need to obtain both parents’ assessments of child adjustment, as the fathers’ assessment was associated with different risk factors compared to the mothers. Four categories of families were distinguished based on the amount and type of disruptions in family rituals and routines, i.e., protecting, emotional disruptive, exposing, and chaotic families (Haugland, 2005). In the present study significant positive correlation between neglecting and object reward with duration of alcohol intake was found. Eiden *et al*. (2004) examined the transactional nature of parent-child interactions over time among alcoholic and non-alcoholic families. Higher paternal alcohol consumption at 12 months was longitudinally predictive of negative parental behavior at 24 months. Results highlighted the nested nature of risk in alcoholic families and the direction of influence from parent to child during interactions and suggested that the pathway to risk among these children is through negative parent-infant interactions. Parents who abuse alcohol were also known to exercise harsh discipline. As described above, alcoholics are easily provoked at the slightest offence. Therefore, they can be excessively harsh and arbitrary in their use of discipline. These forms of discipline can result in the growing alienation of the children from their parents (Windle, 1996).

There were certain limitations of the present study. Firstly, sample size was small. Secondly, only parent-child relationship was seen in the present study. Other areas like family environment and family interaction pattern, behavioral problems in children etc. were not included. Thirdly, response was taken only from the eldest child.

## CONCLUSION

The results showed that the children of alcoholic parents tended to have more symbolic punishment, rejecting, objective punishment, demanding, indifferent and symbolic reward in loving and neglecting than children of non alcoholic parents.
